# The difficulties in cancer treatment

**DOI:** 10.3332/ecancer.2012.ed16

**Published:** 2012-11-14

**Authors:** Sajib Chakraborty, Taibur Rahman

**Affiliations:** University of Dhaka, Dhaka, Bangladesh

## Abstract

Cancer is clearly the most deadly disease in the developed world as one in three people develop cancer during their lifetime. The cure for cancer is like the Holy Grail since most of the existing treatments are not effective enough to provide full protection from this disease. In recent years the burgeoning of sophisticated genomic, proteomic and bioinformatics techniques has made it possible for us to get a glimpse of the intricate interplay of numerous cellular genes and regulatory genetic elements that are responsible for the manifestation of cancerous phenotypes. With the use of modern genomic technologies we are now beginning to understand the enormous complexity of cancer. However there are few success stories as far as the treatment of cancer is concerned. For instance the treatments of leukemia and lymphoma have been established and proved to be satisfactory. Despite occasional successes the treatment for most cancers is still a long way from reality. In this editorial, we have addressed several reasons for the difficulties in cancer treatment.

## Introduction

Cancer or malignant neoplasm is a genetic disorder that results from genetic or epigenetic alterations in the somatic cells. Previous studies showed that tumorigenesis in humans is a multistep process which involves various genetic or epigenetic changes which ultimately drive the malignant transformation of the normal cells. Mutations required for the malignancy can be acquired gradually step by step during various stages of an individual’s lifetime. Apart from the acquired ones, some mutations have hereditary origins and hence are involved in a hereditary form of malignancy such as the familial form of retinoblastoma. Mutations in the cellular proto-onco genes involved in growth and tumour suppressor genes are frequently associated with cancer and hence are considered to be the obvious genetic targets for mutations. Various types of cancer causing genetic aberrations are well characterized such as mutations, gene amplification, translocation, structural deletion, chromosomal miss-segregation etc. For instance the association of Ras mutation with most cancers demonstrates the important role of this oncogene in carcinogenesis. Amplification of c-Myc oncogene and translocation between chromosome 9 and 22 producing BCR-ABL fusion product are involved in malignant transformation.

Apart from the various chemical and physical (ionizing radiation, UV light) carcinogens several biological agents can also contribute to the development of cancer; for example viruses, bacteria and parasites can potentiate a carcinogenic process in humans. Several studies have been directed at characterizing cancer cells. The most significant characterization of cancer cells came from Hanahan and Weinberg. They proposed six essential alterations in cancer cell physiology as a manifestation of the multiple genetic mutations that collectively determine the malignant phenotype. These include self-sufficiency in growth signals, insensitivity to growth-inhibitory (antigrowth) signals, evasion of programmed cell death (apoptosis), limitless replicative potential, sustained angiogenesis, and tissue invasion with metastasis [1]. With the aim of minimising the incidence of cancer, several potential risk factors such as diet, lifestyle, smoking, alcoholism, viral infection etc. for cancer has been identified. Among these the most significant association was found between lung cancer and smoking.

## Reasons for the difficulties in cancer treatment

### 1. Targeting cancer stem cells (CSCs) is difficult

The growing body of evidence suggests that in many cases cancerous cells originate from a single cell with stem cell characteristics. These findings should have a profound effect on the treatment of cancer. Traditional cancer treatment is based on the assumption that all somatic cells possess a similar malignant potential. The lack of specificity in these strategies has made them ineffective to provide long lasting protection against cancer. In contrast the drugs that are more target-specific can cause the regression of the bulk of the tumour but in most cases fails to eliminate the cancer stem cells and results are proved to be devastating as the recurrence of the tumour is commonly observed after the discontinuation of the drug administration. The most prominent example is Gleevec (Imatinib) used in Chronic Myelogenous Leukemia (CML). It acts via targeting the BCR-ABL fused protein which has tyrosine kinase activity. This drug interferes with the intracellular growth signaling pathway in cancer cells by blocking the ATP binding site of BCR-ABL and thus prevents it from transducer signalling to its target protein. Although this drug has proved to be very effective it lacks curative potential as the recurrence of the tumour has been observed after the discontinuation of the medication. The persistence of philadelphia chromosome positive stem cells was held to be responsible for this phenomenon [2].

So the cancer stem cell hypothesis will be crucial to our understanding of cancer biology and can dictate the course of future therapeutic strategies. There are some barriers to be overcome in order to identify and target cancer stem cells as the research into cancer stem cells is still in its early days. For example it is not clearly known whether a progenitor cancer cell acquires the ability to self-renew. Moreover the mutations that are involved in the self-renewal process are still unidentified. To overcome this problem detailed studies should be directed to reveal the biology of cancer stem cells. The transcriptome profiling of the cancer initiating cells and their progeny could unveil some interesting aspects and ultimately could lead to the identification of some genes which are associated with the stem cell characteristics of cancer initiating cells.

### 2. Drug resistance properties of cancer stem cells make them immune to anticancer drugs

Since normal stem cells have to undergo a repeated process of self-renewal and differentiation in the entire life span of an individual, they have developed certain unique mechanisms by which they can protect themselves from harmful xenobiotic agents. For example ATP-binding cassette (ABC) transporter proteins tend to be expressed at high levels in stem cells. These transporter proteins can utilize the energy released from ATP hydrolysis to facilitate an energy dependent substrate efflux which ultimately helps the stem cells to flush out toxic agents from the intracellular cytoplasm to the extracellular space against the concentration gradient. Cancer stem cells are believed to maintain this property since different types of drug resistant cancer cells were found to express this protein. The most notable example is the drug resistance protein expressed in breast cancer cells - Breast Cancer Resistance protein (BCRP) ABCG2, a specific ATP-binding cassette transporter. This protein was previously identified in a variety of stem cells and is responsible for the drug efflux in breast cancer. The BCRP-ABCG2 protein was also found to be highly expressed in CD34^+^/CD38^-^ cells, considered as a stem cell subpopulation in Acute Myelogenous Leukemia (AML) and was actively involved in the drug efflux [3]. CSCs can also adopt alternative strategies to confer drug resistance. For example the proposed tumour initiating cells (CD34^+^ cells) in AML highly express aldehyde dehydrogenase (ALDH) which is commonly expressed in hematopoietic stem cells. ALDH typically acts as a detoxifying enzyme that confers resistance to alkylating agents such as cyclophosphamide.

The current understanding of cancer stem cell biology indicates that CSCs may be a rare cell population and only comprise a very tiny proportion of tumour cells. They are also believed to be quiescent. This poses another problem in targeting the CSCs by conventional chemotherapeutic drugs which exert their toxicity to rapidly dividing cells. As the CSCs are not involved in vigorous cell division process they are less likely to be affected by chemotherapeutic agents.

### 3. Lack of cancer epigenetic profiling and specificity of existing epi-drugs

The traditional approach in cancer research was primarily focused on the identification and determining the general patterns of genetic anomalies that result from the mutational or other chromosomal aberration events. But unfortunately only a handful of genetic mutations associated with cancer have been identified as among patients which by no means explains the enormous genetic deviation that eventually manifests in the malignant phenotype of cancer. Our current knowledge of the epigenetic mechanism could fit in to this puzzle and could prove to be the missing link. It has been shown that epigenetic mechanisms can promote the initiation of tumorigenesis and can facilitate the persistence of the malignant phenotype in cancer cells [4]. Several studies were conducted with the aim of identifying the specific epigenetic changes that contribute to cancer development. For example both promoter hypermethylation, responsible for transcriptional inactivation of genes, and genome-wide hypomethylation causing a genomic instability have been observed in cancer. Methylation in CpG island residing in the promoter region of the tumour suppressor genes such as Rb, p16 and p53 could have a disastrous effect on cell cycle regulation. The silencing of these genes can lead to the uncontrolled proliferation of cells. The enzyme responsible for the methylation of CpG dinucleotide is known as DNA methyl transferase (DNMT). This enzyme is the obvious target for reestablishing the normal methylation pattern in the cis elements of the affected gene. In harmony with this notion several DNMT blockers were identified including 5-aza-2′-deoxycytidine which successfully diminished the tumorigenicity in a mouse model of neoplasia [5]. But the concept of targeting the DNMT gives rise to the concern regarding its lack of specificity. This is because, as the inhibiting agents are not specific for the affected hypermethylated genes (eg. tumour suppressor genes), they can lead to hypomethylation of the whole genome. This will subsequently activate some other genes randomly which are supposed to be silenced in an adult individual.

So there is an urgent need to develop a specific epigenomic drug which is still far from the current situation. Apart from the promoter hypermethylation, there is another type of epigenetic aberration involving the methylation pattern which is associated with cancer cells. The cancer cell genome is largely hypomethylated compared to normal cells. And the use of the DNMT blocker could actually turn the situation into something much worse as it will further decrease the methylation level which may lead to activation of the previously silenced oncogene. Moreover methylation/demethylation is not the only epigenetic mechanism that regulates the differential expression of genes. For instance histone mofication by histone acetylase/deacetylase and histone methylase/demethylase also contribute to epigenetic regulations. Both the histone lysine acetylation and methylation are observed in cancer cells. The variation in histone modification patterns are not only observed in different types but also in different stages of the same cancer. So understandably the current picture of the total epigenetic signature of different types of cancer is still obscure and certainly demands further research. The whole epigenomic map for all human chromosomes can certainly help the researcher to establish a epigenetic pattern for cancer cells. Cancer epigenomic profiling could be used not only for the development of novel drugs but also could facilitate the early diagnosis of cancer which is also regarded as a major problem for treatment.

### 4. Problems associated with cancer diagnosis make it difficult to treat

The non-specific nature of cancer symptoms makes diagnosis difficult. In certain cases the patient remains asymptotic. So these early signs and symptoms of cancer are often neglected by the patient which provides the opportunity for the cancer to spread without any medical intervention. By the time the patient seeks medical help, it may be out of reach of available clinical treatment. Some examples of the diagnosis difficulties of certain cancers are given below:

#### a) Oesophageal cancer

Oesophageal cancer is one of the most lethal cancers and it is difficult to treat. Unfortunately early detection of this cancer is very difficult simply because in the early phase of this cancer smaller tumours often cause few or no symptoms. But if undetected oesophageal cancer can spread into various parts of the body including the stomach, lungs, liver and lymph nodes. In the late metastasized stage the tumour is incurable and most of the treatment of late stage only focuses on extending life and relieving the symptoms.

#### b) Prostate cancer

Prostate cancer tends to occur in older people who are aged over fifty. It is one of the most prevalent cancers in older males. Like oesophageal cancer, the patient may not show any symptoms in the early stage. Since prostate cancer in most cases is slow growing and symptom free it remains undetected and often metastasizes from the prostate to different parts of body especially to the bones and lymph nodes. The presence of prostate cancer may be diagnosed by PSA (Prostate specific antigen) or biopsy. However there is some controversy about the specificity of the PSA test. Suspected prostate cancer is better to confirm by biopsy.

#### c) Pancreatic cancer

Pancreatic cancer is called the “silent” disease because it does not often show early symptoms and also in the later stages patients with pancreatic cancer show non-specific symptoms. Moreover the symptoms tend to vary and may depend upon the location of the cancer.

### 5. Unavailability of effective biomarkers for cancer diagnosis and prognosis

The unavailability of good biomarkers is another hindrance for cancer treatment. Biomarkers are not only important for diagnostic purposes but can also be of great prognostic value. With the identification of the right biomarker the cancer progression and effect of chemotherapeutic drugs can be evaluated in great detail. But unfortunately the hunt is still on to identify reliable biomarkers for different cancers. The recently emerged concept of clinical proteomics has shown much promise in identifing effective biomarkers that would enable early detection and the disease progression of cancer. But there are some technical difficulties in using proteomic profiling as a diagnostic tool which means that further research, standardization and validation of this approach is needed.

### 6. Limitations of conventional chemotherapeutic agents

The existing chemotherapeutic drugs are toxic to all cells including cancer and normal cells. So the administration of these toxic agents kill the rapidly proliferating cancer cells as well as the normal cells which may lead to some serious side effects and may sometimes cause the death of patients. Untargeted radiotherapy suffers from a similar lack of specificity.

### 7. Metastasis poses a huge problem in cancer treatment

One of the main reasons for the difficulties associated with cancer treatment is the metastatic nature of cancer. The asymptomatic nature of certain cancers and the lack of diagnosis allow the cancer to spread to different parts of the body from its site of origin without any medical intervention. The first site where the cancer is starts is called the “primary cancer site” whereas the sites in which cancer has spread is known as the “secondary or metastatic site”. In order to spread the cancer cells, primary sites have acquired the ability to invade and colonize a distant site and eventually spread into different parts. There are three major methods of cancer metastasis: local spread, through blood circulation and via the lymphatic system. So when cancer metastasizes the treatment should not only be directed towards the primary cancer but also needs to eliminate the secondary ones. This poses a great problem. Moreover there are certain metastatic events in cancer which are too small to be detected. These are called micrometastases events. For a few cancers, blood tests can detect the marker proteins released by the cancer cells. These markers can indicate the presence of cancer spread which is difficult to identify by normal scanning. But unfortunately most of the cancer specific markers have not yet been identified.

## Conclusion

By solving the identified reasons for the difficulties in cancer treatment we can reduce the incidence of different types of cancer.
